# N, P, and S Codoped Graphene‐Like Carbon Nanosheets for Ultrafast Uranium (VI) Capture with High Capacity

**DOI:** 10.1002/advs.201800235

**Published:** 2018-08-27

**Authors:** Zhe Chen, Wanying Chen, Dashuang Jia, Yang Liu, Anrui Zhang, Tao Wen, Jian Liu, Yuejie Ai, Weiguo Song, Xiangke Wang

**Affiliations:** ^1^ College of Environmental Science and Engineering North China Electric Power University Beijing 102206 P. R. China; ^2^ Laboratory of Molecular Nanostructures and Nanotechnology Institute of Chemistry Chinese Academy of Sciences Beijing 100190 P. R. China

**Keywords:** adsorption, density functional theory (DFT) calculations, graphene, heteroatoms, uranium

## Abstract

The development of functional materials for the highly efficient capture of radionuclides, such as uranium from nuclear waste solutions, is an important and challenging topic. Here, few‐layered N, P, and S codoped graphene‐like carbon nanosheets (NPS‐GLCs) that are fabricated in the 2D confined spacing of silicate RUB‐15 and applied as sorbents to remove U(VI)ions from aqueous solutions are presented. The NPS‐GLCs exhibit a large capacity, wide pH suitability, an ultrafast removal rate, stability at high ionic strengths, and excellent selectivity for U(VI) as compared to multiple competing metal ions. The 2D ultrathin structure of NPS‐GLCs with large spacing of 1 nm not only assures the rapid mass diffusion, but also exposes a sufficient active site for the adsorption. Strong covalent bonds such as P—O—U and S—O—U are generated between the heteroatom (N, P, S) with UO_2_^2+^ according to X‐ray photoelectron spectroscopy analysis and density functional theory theoretical calculations. This work highlights the interaction mechanism of low oxidation state heteroatoms with UO_2_^2+^, thereby shedding light on the material design of uranium immobilization in the pollution cleanup of radionuclides.

## 1 Introduction

The annual development of nuclear power plants is intensive and widespread around the world. As a result, the prevention of radionuclide release to the environment and reduction of human exposure to radiation has become increasingly important. Uranium, which may come from uranium mining, nuclear fuel production, and even nuclear accidents, is one of the dominant components of nuclear wastes. Uranium can exist in a variety of valence states, and the uranyl cation (UO_2_^2+^) that dissolves in water is the most common and important species in nature.^1^ Therefore, research attention has been focused on the development of functional materials to clean these radionuclides from contaminated water and soil.

Among the different methods for uranium removal from nuclear waste, such as liquid–liquid extraction,^2^ ion exchange,^3^ adsorption,^4^ and reductive precipitation,^5^ adsorption is a popular method due to its high efficiency, convenient operation, and low cost. Various kinds of adsorbent material have been developed to efficiently remove and recover radionuclides, including traditional adsorbent such as clay minerals,^6^ nanoscale zero‐valent iron,^7^ and layered double hydroxides (LDHs).^8^ However, clays and LDHs often suffer from slow adsorption kinetics, limited selectivity, and low adsorption capacity. At the same time, novel adsorbents are emerging, such as layered metal sulfides,^9^ graphene oxides (GO), porous organic polymers,^10^ metal–organic frameworks (MOFs),^11^ and covalent organic frameworks (COFs). Wang and co‐workers reported the amidoxime appended metal–organic framework UiO‐66‐AO for rapid and efficient extraction of uranium from seawater, which forming strong interaction between amidoxime ligands and uranyl (VI) ions.^12^ Ma and co‐workers designed the amidoxime‐functionalized COFs, and proved it highly efficiency in the U extraction from spiked seawater with capacity of 127 mg g^−1^.^13^ These results broaden the adsorbent material types, supplied insight between highly reticular/topological structure with chelating groups and uranyl (VI) ions.

The adsorption performance of graphene, GO, and their composite materials has also been investigated in the removal of radionuclides.^14^ Generally, graphene produced from mechanical exfoliation or chemical vapor deposition exhibits an unsatisfying radionuclides adsorption performance due to the absence of functional groups on the surface. Meanwhile, GO nanosheets, which contain abundant carboxyl (—COOH) groups and hydroxyl (—OH)/epoxy groups (—O—) possess good prospect for the application of radionuclide pollution treatment.^15^ Wang and co‐workers prepared few‐layered GO nanosheets and found that the abundant carboxyl groups/hydroxyl groups on the GO nanosheets were essential in U(VI) sorption.^16^ However, the electrostatic repulsion of the carboxyl (—COOH) groups with uranyl often resulted in limited adsorption capacity, slow adsorption kinetics, and a high pH dependence. Apart from GO, some phosphorus and sulfur compounds have also been proved to be efficient in U(VI) retention.^17^ Lin synthesized phosphoryl urea‐derived MOFs with a high capacity for uranyl adsorption, and inferred that it interacted with uranium through the P=O group at appropriate distances inside the MOF cavities. On the other hand, sulfides were reported to be effective for UO_2_^2+^ capture due to the presence of strong UO_2_^2+^···S^2−^ bonding interactions.^9^, ^18^ With the exception of oxygen‐containing functional groups, the application of graphene‐containing heteroatoms (N, P, S, etc.) as sorbents in the removal of radionuclides from aqueous solution is rare, which may be due to the difficulty of synthesizing high‐quality and uniformly doped graphene. As a result, the development of a method for the fabrication of heteroatom‐doped graphene and the investigation of its adsorption performance and complexation mechanism is highly significant scientifically.

In this work, we are aimed at (1) develop a facile method to prepare few‐layered N, P, and S codoped graphene‐like carbon nanosheets (denoted as NPS‐GLCs) in confined 2D spacing of silicate RUB‐15. The heteroatoms were doped into graphene lattice instead of grafting on graphene surface; (2) apply the NPS‐GLCs as sorbents to remove U(VI) ions from aqueous solutions and investigate the capacity, the kinetics, selectivity, the effect of pH and ionic strength on U(VI) sorption; (3) discuss the interaction mechanism of UO_2_^2+^ and NPS‐GLCs with X‐ray photoelectron spectroscopy (XPS) and theoretical calculation. This study developed a delicate method to fabricate heteroatom (N, P, S, etc.) doped graphene‐like ultrathin carbon nanosheets, and also demonstrated the superior adsorption ability and interaction mechanism of UO_2_^2+^ on heteroatom. We observed the maximum capacity (*Q*_m_) of 294.16 mg g^−1^ on NPS‐GLC, along with pH effective range 5–9, ultrafast removal rate (80.5% removal percentage achieved at 5 min), durability in high ionic strengths, and high selectivity toward competing metal ions. All the features make it potential applicability of uranium immobilization in environmental pollution cleanup.

## 2 Results and Discussion

**Figure 1:**
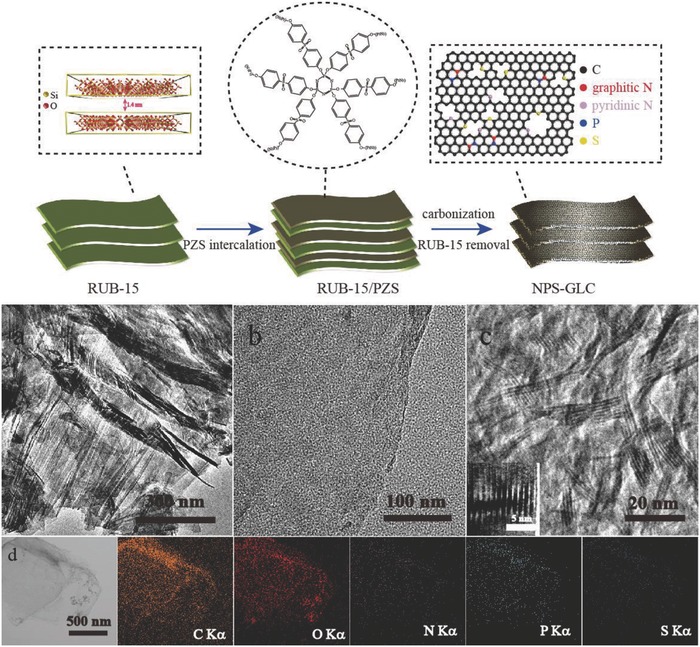
The fabrication route of NPS‐GLCs. a–c) TEM and HRTEM images of NPS‐GLCs; d) Compositional EDS mapping of NPS‐GLCs.

The synthesis of the NPS‐GLCs is illustrated in **Figure** **1**. First, the RUB‐15 nanosheets were prepared following a previous report. As shown in Figures S1 and S2 (Supporting Information), RUB‐15 ([N(CH_3_)_4_]_8_[Si_24_O_52_(OH)_4_]·20H_2_O) is an aluminum‐free layered silicate and a zeolite precursor with zeolitic framework composed of a halved sodalite cage. The X‐ray diffraction (XRD) pattern of RUB‐15 had a strong signal at 2θ = 6.32°, thereby suggesting a basal spacing of 1.40 nm (Figure S3, Supporting Information). RUB‐15 exhibited a layer spacing of 1.4 nm, thereby indicating that the diffusion of two reagents, namely, hexachlorocyclo‐phosphazene and 4,4′‐sulfonyldiphenol, was favored in the synthesis of highly cross‐linked poly(cyclotriphosphazene‐*co*‐4,4′‐sulfonyldiphenol) (denoted as PZS). With the catalysis of triethylamine, the PZS shells were generated through the in situ polymerization of the phosphonitrilic chloride trimer and 4,4′‐sulfonyldiphenol in each individual layer spacing of the RUB‐15 nanosheets. Next, RUB‐15/PZS nanosheets were calcined in Ar atmosphere, thereby resulting in the carbonization of the PZS layer. During the calcination process, the nitrogen, phosphorus, and sulfur atoms in the PZS were uniformly doped into the graphene lattice of the carbon nanosheets. Finally, the RUB‐15 nanosheets were removed by corrosion in the presence of NaOH solution and NPS‐GLC yielded as last.

The produced NPS‐GLCs exhibited a graphene‐like single layer or very few layers of carbon nanosheets given that polymerization and carbonization were executed in the confined interlamination of RUB‐15. The typical transmission electron microscopy (TEM) images of the NPS‐GLCs are presented in Figure 1a–d. Unlike the reduced graphene oxide (RGO) produced by the Hummer method, the NPS‐GLC exhibited wrinkled appearance was composed of several graphene‐like carbon lamellas that were stacked and highly aligned. A single layer or very few layers of graphene‐like ultrathin carbon nanosheets are presented in Figure 1b. In addition, a uniform quasi‐lamellar structure (Figure 1c) was observed in many areas of the TEM image. At an angle with a perfect view, the layer spacing of NPS‐GLCs was measured to be 1.0 nm (inset of Figure 1c), which strongly validated the generation of the NPS‐GLCs in the interlamination of RUB‐15 given that the layer spacing of the NPS‐GLCs was a result of the removal of RUB‐15. The RUB‐15 nanosheets played an important role as the hard template, consisting with previous report by Zhu et al.^19^ Figure S4 (Supporting Information) displayed the atomic force microscopy (AFM) images of NPS‐GLCs, proving the mophology of few layers of ultrathin graphene‐like membranes. The cross‐section height profile revealed a small thickness of 1.0 nm, that was, two or three graphitic layers, which agreed well with the TEM images. The energy‐dispersive spectroscopy (EDS) mapping of the NPS‐GLCs by use of scanning transmission electron microscopy (STEM) (Figure 1d) revealed the homogeneous distribution of the nitrogen, phosphorus, and sulfur atoms throughout the NPS‐GLCs. As the heteroatoms dispersed highly ordered in PZS, we could predict that N, P, and S were well‐distributed in the NPS‐GLCs following thermal calcination. Because the N, P, and S were doped accompany the recombination of the carbon atoms into graphene during annealing. As a result, the substitutional multi‐heteroatom could also be in situ doped into the graphene lattice. The method supplied homogeneous and highly dispersed heteroatoms on graphene, which is generally difficult to produce by traditional doping methods.^20^ The elemental analysis of the NPS‐GLCs further confirmed the existence of N, P, and S in the NPS‐GLCs, with the contents of 2.32, 2.85, and 0.95 at%, respectively.

**Figure 2:**
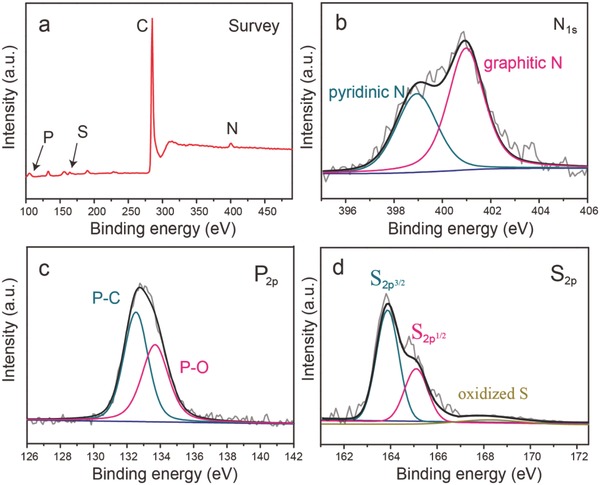
XPS spectra of a) full spectrum survey, b) N1s, c) P2p, and d) S2p of the NPS‐GLCs.

The XPS analysis was performed to characterize the state of the nitrogen, phosphorus, and sulfur atoms in the NPS‐GLCs. The high‐resolution N 1s spectrum of the NPS‐GLC (**Figure** **2**b) was divided into two peaks at 398.9 eV (pyridinic N) and 401.0 eV (graphitic N). The high‐resolution P 2p spectrum exhibited two peaks at ≈132.4 and 133.6 eV, which corresponds to P—C and P—O, respectively (Figure 2c). The S 2p spectrum presented three peaks at 163.8, 165.1, and 168.2 eV, which were ascribed to the 2p^3/2^ and 2p^1/2^ splitting of the S 2p spin orbital (—C—S—C—) and the oxidized S, respectively (Figure 2d).^21^ The XPS analysis confirmed the successful incorporation of the N, P, and S atoms onto the NPS‐GLCs, as well as the reduction of a large proportion of P and S from PZS to a low oxidation state.

**Figure 3:**
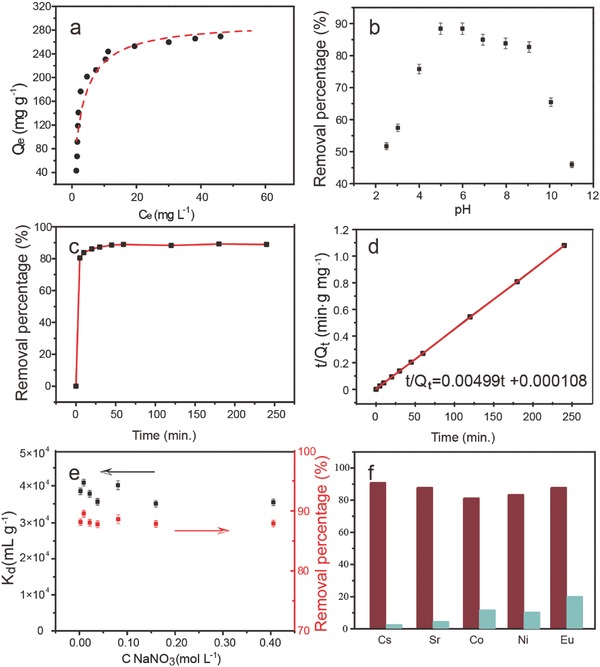
a) Adsorption isotherm of U(VI) on NPS‐GLCs; b) Adsorption curve of U(VI) on the NPS‐GLCs as a function of pH; c) Time‐dependent removal of U(VI) on the NPS‐GLCs (initial U(VI) concentration of 20 mg L^−1^); d) The kinetics plot of *t*/*Q*_t_ versus time fitted with the pseudo‐second‐order kinetic model. e) *K*_d_ as a function of the concentration of NaNO_3_ (black) and the removal percentage as a function of the NaNO_3_ concentration (red); f) Sorption percentages of U(VI) (red) and the competitive metal ions (blue) in the binary‐metal systems.

The resulting NPS‐GLCs were applied to remove U(VI) from the aqueous solutions to explore its adsorption performance. The adsorption isotherm of U(VI) on the NPS‐GLCs was obtained at different initial concentrations ranging from 5 to 100 mg L^−1^ at a pH of 5. As clearly noted in **Figure** **3**a, the adsorption amount of U(VI) grew successively following an increase in the initial U(VI) concentration. The adsorption data of the NPS‐GLCs for UO_2_^2+^ were fitted well with the Langmuir and Freundlich models, and the corresponding parameters are tabulated in Table S1 (Supporting Information). The adsorption isotherm of U(VI) on the NPS‐GLCs was better fitted by the Langmuir model (*R* ^2^ > 0.993) than the Freundlich model (*R* ^2^ > 0.765), with a maximum capacity (*Q*_m_) as high as 294.16 mg g^−1^. The Langmuir model characterized the adsorption process as a monolayer chemical adsorption or ion exchange. The maximum capacity was higher than that of graphene oxide (27.61 mg g^−1^),^22^ graphene/iron oxides composite (69.49 mg g^−1^),^23^ or conventional inorganic materials such as activated carbon (28.30 mg g^−1^),^24^ pyrrhotite (21.34 mg g^−1^),^25^ and LDH/GO composite (129.87 mg g^−1^).^26^

Meanwhile, the N, P, and S codoped carbon bulk materials were synthesized by the same approach as the NPS‐GLCs except that no layered silicate RUB‐15 was used as template. The adsorption isotherm for U(VI) is shown as Figure S6 (Supporting Information). Compared with the NPS‐GLCs, the bulk materials exhibited much lower maximum capacity of only 83 mg g^−1^ at pH = 5, proving that the 2D ultrathin structure of NPS‐GLCs not only assured the rapid mass diffusion, but also exposed sufficient active site for the adsorption.

The surface charge/active sites of the sorbent and the speciation of U(VI) were different at different pH, so the pH value greatly influenced on the adsorption behavior of U(VI). As presented in Figure 3b, the adsorption capacity of U(VI) on NPS‐GLCs rose rapidly following an increase in the pH value from 2 to 5. It exhibited a very high percentage of uranium removal (>82%) between a pH of 5 and 9, which indicated its widespread availability in either weakly acidic, weakly alkaline, or neutral water systems. A maximum U removal of 88.5% was achieved at a pH of about 5. In brief, the wide suitability at a pH between 5 and 9 endowed NPS‐GLCs highly prospect in waste water remediation.

The adsorption kinetics of U(VI) on the NPS‐GLCs was investigated at an initial U(VI) concentration of 20 mg L^−1^ and a pH of 5 (Figure 3c). Obviously, the adsorption of U(VI) onto the NPS‐GLCs was an ultrafast process. About 80.5% of U(VI) was rapidly generated at the first contact time of 5 min. The amount of U(VI) removal grew slowly and reached equilibrium of 89.0% after 30 min. Following the detailed simulation and analysis, the kinetic data of the adsorption of U(VI) on the NPS‐GLCs were characterized to perfectly fit pseudo‐second‐order kinetic model (Equation (3) in Method section of the Supporting Information). The plot of *t*/*Q*_t_ versus *t* (Figure 3d) exhibited a perfectly linear relation (correlation coefficient *R* ^2^ = 1). From the kinetic equation, *Q*_e_ and *k* were calculated to be 200.4 mg g^−1^ and 0.231 g mg^−1^ min^−1^, respectively. Previous studies reported pseudo‐second‐order kinetic models for the adsorption of U(VI) by other materials such as GOs,^27^ amidoximated magnetite/GO composites,^28^ FeS,^29^ and melamine‐modified graphene hydrogels.^30^ The values of *k* were much higher than those previous references, which confirmed the significant adsorption efficiency of the NPS‐GLCs. From the pseudo‐second‐order kinetic model, the rate‐limiting step of the adsorption of U(VI) on the NPS‐GLCs was characterized as chemical adsorption or strong surface complexation rather than mass transport.^31^

The selective removal of radionuclides in the presence of different salts at high concentrations from industrial nuclear waste was requisite. The performances of the NPS‐GLCs for U(VI) adsorption at a high ionic strength were tested and shown in Figure 3e. In general, a material with a *K*_d_ value reaching 10^4^ mL g^−1^ was considered as an excellent adsorbent.^32^ The removal percentage of U(VI) ranged from 87.5 to 89.2%, and the *K*_d_ values varied from 3.50 × 10^4^ to 4.13 × 10^4^ mL g^−1^ in a competitive adsorption experiment containing NaCl (concentration ranged from 0.00 to 0.40 mol L^−1^) and 10 mg L^−1^ U(VI). The removal percentage was almost unchanged following an increase in the NaNO_3_ concentration. Therefore, it was evident that the NPS‐GLCs maintained excellent U(VI) adsorption despite the presence of a tremendous excess of NaNO_3_ (Na/UO_2_^2+^ molar ratio = 5120). The adsorption process was independent of the ionic strength, thereby proving that the adsorption behavior was dominated by inner sphere surface complexation.^33^

Many metal ions existed in the wastewater in actual application, and these ions could compete with U(VI) for binding on the NPS‐GLCs surface sites. To explore the selectivity of the NPS‐GLCs, the competitive adsorption on U(VI) in the presence of competing metal ions (Cs^+^, Sr^2+^, Co^2+^, Ni^2+^, and Eu^3+^) were, respectively, investigated at a pH of 5.0 and in the presence of equimolar U(VI) and competing ions. Figure 3f was the histogram of the removal percentage of U(VI) and the competing metal ions by the NPS‐GLCs. The removal percentage of U(VI) varied from 82.0 to 90.5%, which indicated its independence from the coexisting Cs^+^, Sr^2+^, Co^2+^, Ni^2+^, and Eu^3+^ ions. The maximum U(VI) removal of 90.5% and minimum U(VI) removal of 82.0% were generated in the U + Cs and U + Co binary‐metal systems, respectively. Extremely low sorption percentages Cs^+^, Sr^2+^, Co^2+^, and Ni^2+^ ions (<10%) were simultaneously obtained on the NPS‐GLCs, though 19.8% Eu^3+^ was adsorbed. The adsorption of Eu^3+^ might be increased due to its relatively high ionic charge with the surface sites of the NPS‐GLCs.^34^ The removal percentages of U(VI) and the metal ions as well as the calculated *K*_s_(U/M) values are represented in Table S2 (Supporting Information). The high *K*_s_(U/M) value revealed the superior capability of the NPS‐GLCs for the selective preconcentration of U(VI) from the multicomponent wastewater. According to the changing trend of the *K*_s_(U/M) values, the NPS‐GLCs exhibited a selective sorption order: UO_2_^2+^ > trivalent Eu^3+^ > divalent transition metal ions > divalent alkaline earth metal ions > monovalent alkaline metal ion (Table S2, Supporting Information). Generally speaking, the metal cations interacted with the adsorbent through electrostatic or van der Waals force. No functional groups with a strong electrostatic force that could combine with UO_2_^2+^ existed on the graphitized NPS‐GLCs, which decided the relatively poor adsorption of Cs^+^, Sr^2+^, Co^2+^, and so on. On the other hand, stronger affinities, such as covalent bonds, were the key forces between the NPS‐GLCs and UO_2_^2+^, and that was why the competitive ions had little depression impact on the sorption of UO_2_^2+^. The ignorable effects of the competitive ions to the adsorption of UO_2_^2+^ proved that the interaction between UO_2_^2+^ and the NPS‐GLCs was not electrostatic force or van der Waals force but covalent bonds, which was very strong even in the presence of high concentrations and high ionic‐charged competitive ions in solutions. This deduction was also verified in the following XPS analysis and density functional theory (DFT) calculations.

**Figure 4:**
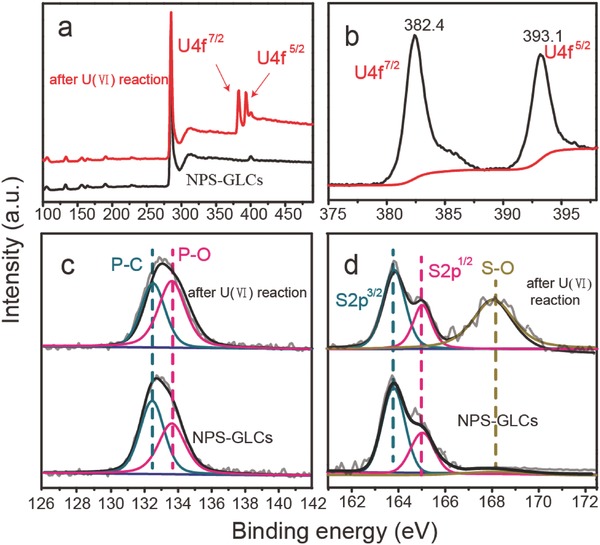
X‐ray photoelectron spectra of a) the survey, b) uranium, c) phosphorus, and d) sulfur spectra of the NPS‐GLCs and UO_2_^2+^ ion‐adsorbed NPS‐GLCs.

The X‐ray photoelectron spectra of the survey spectra, uranium, phosphorus, and sulfur spectra of the NPS‐GLCs before and after U(VI) adsorption are shown in **Figure** **4**. According to the survey spectra in Figure 4a, the U(VI)‐adsorbed product maintained all the peaks in the NPS‐GLCs except for two evident peaks at 382.4 and 393.1 eV, which correspond to newly generated U 4f^7/2^ and U 4f^5/2^, thereby indicating that a significant number of U(VI) was adsorbed by the NPS‐GLCs.^35^ The P—O peaks at 133.6 eV and S—O at 168.2 eV in the UO_2_^2+^‐adsorbed NPS‐GLCs increased remarkably as compared to the bare NPS‐GLCs, suggesting that the P and S were oxidized along with UO_2_^2+^ adsorption.^36^ We maintained that the UO_2_^2+^ ions were fixed on the NPS‐GLCs through the strong covalent bonds of P—O—U and S—O—U. Following the reaction with U(VI), the chemical bonds of P—O—U and S—O—U promoted the electron cloud shift from P (or S) toward the O atoms. As a result, the P and S atoms were oxidized.

**Figure 5:**
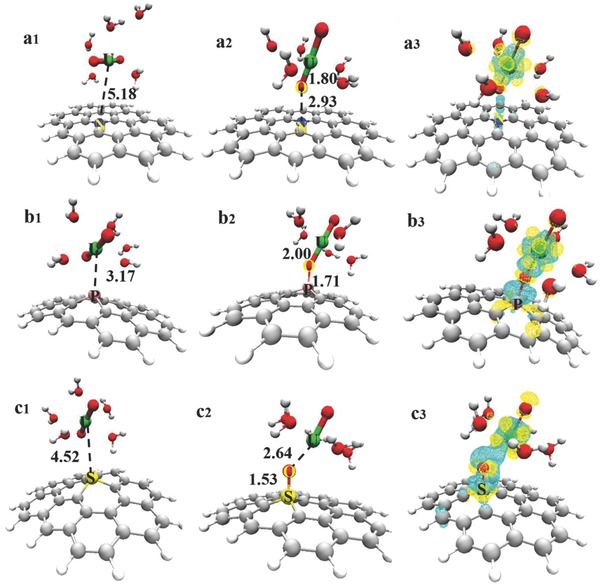
a1–c1) Optimized structures of the G‐X/Uranyl (U) complexes. a2–c2) Optimized structures of the G‐X/uranyl (O) complexes. a3–c3) Charge density difference plot of the uranyl ion absorbed on the graphene surface substituted with the X atom, where X represents N, P, and S atoms, respectively. The yellow regions represent the electron accumulation and the light blue regions represent the electron deficiency. The isosurface has a value of 0.06 e Bohr^−3^.

The DFT was applied to understand the corresponding interaction mechanism of the N, P, and S‐doped GLCs with uranyl ions. A finite ten‐carbon ring model (Figure S9, Supporting Information) was chosen for graphene considering both its computational cost and accuracy. The optimized configurations of the different G‐X/uranyl complexes are presented in **Figure** **5**. The present study examined two possible interaction sites, specifically G‐X/uranyl (U) and G‐X/uranyl (O), for each X element‐doped graphene complex.

For N‐doped graphene, the N—O distance (2.93 Å) was much shorter than the N—U distance (5.18 Å), thereby indicating the presence of a higher interaction energy, which is confirmed in Table S3 (Supporting Information). At the same time, situations for the P‐doped and S‐doped graphene complexes exhibited quite different. The optimized structures of the G‐P/uranyl complexes are shown in Figure 5b1,2, wherein physical adsorption (*E*_ad_ = 9.78 kcal mol^−1^) was observed between the U and P atoms at a bond distance of 3.17 Å. However, chemical adsorption was observed in the G‐P/uranyl (O) configuration at a strong P—O bond length of 1.71 Å. The adsorption energy also greatly increased to 19.33 kcal mol^−1^. For S‐doped graphene, physical adsorption was observed in the G‐S/uranyl (U) complex at an adsorption energy of 7.26 kcal mol^−1^. Meanwhile, a new strong S—O bond length was generated in the G‐S/uranyl (O) complex, as presented in Figure 5c2, which weakened the original U—O bond in uranyl. In summary, the main effective adsorption site of the NPS‐GLCs toward UO_2_^2+^ was located in the X—O interaction, which introduced a strong chemical adsorption process to achieve stable and fast adsorption. Further evidence was derived from the charge density difference plots of the G‐X/uranyl (O) complexes, as presented in Figure 5a3–c3. The charge density difference distributions indicate the charge density changes following the adsorption of UO_2_^2+^ to the graphene surfaces. Unlike the G‐N/uranyl (O) complex, certain obvious charges were transferred near the G‐P/uranyl (O) and G‐S/uranyl (O) interfaces. Such charge transfers facilitated in different atomic binding near the interface. In addition, the uranyl group donated a small amount of electrons to the G‐P substrate, to which more electrons accumulated, as presented in Figure 5b3. Such electron density redistributions on the graphene surface and the effective charge transfer between the uranyl ion and graphene promoted uranyl adsorption. Briefly, the efficient sorption of UO_2_^2+^ on the NPS‐GLCs was mainly due to the formation of strong covalent bonds, such as P—O—U and S—O—U, as well as the presence of high surface energy sites and reducibility that was derived from the defects and holes that were abundantly present in the NPS‐GLCs (see Figure S10, Supporting Information).

## 3 Conclusions

In conclusion, we fabricated few‐layered NPS‐GLCs in aid of the 2D confined spacing of silicate RUB‐15. Compared with common carbon adsorbent such as graphene oxide, the prepared materials in this manuscript exhibited larger capacity, faster adsorption rate, and broader pH range. In addition, the selective adsorption of uranium in the presence of other ions on the NPS‐GLCs was also attractive. The special composition and structure were responsible to the superior performance: (1) the method supplied homogeneous and highly dispersed heteroatoms on GLCs. Different from traditional modification method, the substitutional heteroatom was in situ doped into the graphene lattice, endowing it chemical and performance stability. (2) The 2D packed structure with a spacing of 1 nm exhibits exceptional accessibility for the active sites, allowing significantly higher utilization efficiency. In addition, the 2D layers packed parallel to each other closely, which may promote the cooperation between the heteroatoms bilevel, thereby leading to high affinity toward UO_2_^2+^. The XPS analysis was well in agreement with the DFT calculations, thereby validating that the strong covalent bonds between P—O—U and S—O—U as well as the reducibility from the defects and holes on the NPS‐GLCs were the key active sites for U(VI) fixation. This work shed light on the interaction mechanism of low oxidation state heteroatoms with U(VI) and aided in designing an adsorbent for the immobilization of uranium in the pollution cleanup of radionuclides.

## 4 Experimental Section

*Materials*: TEOS (tetraethyl orthosilicate), TMAOH (tetramethylammonium hydroxide, 25 wt% solution), hexachlorocyclo‐phosphazene (98%), 4,4′‐sulfonyldiphenol (99%), and triethylamine were all provided by Alfa‐Aesar. The RUB‐15 was synthesized by hydrothermal method as described in previous references.^37^ RUB‐15 (200 mg) were dispersed in 20 mL of methanol solution containing 300 mg of hexachlorocyclophosphazene and 675 mg of 4,4′‐sulfonyldiphenol. After stirring for 5 min, 0.74 mL of triethylamine was added dropwise and the solution was stirred for 6 h. Then the precipitates RUB‐15@PZS were collected and washed with methanol three times and dried in vacuum at room temperature for 12 h. In the last step, the RUB‐15@PZS was calcined at 700 °C for 4 h in a tubular furnace under Ar atmosphere, and the NPS‐GLCs were obtained after RUB‐15 removing with NaOH solution corrosion.

*Synthesis of NPS‐GLCs*: RUB‐15 (200 mg) was dispersed in 20 mL of methanol solution containing 300 mg of hexachlorocyclophosphazene and 675 mg of 4,4′‐sulfonyldiphenol. After stirring for 5 min, 0.74 mL of triethylamine was added dropwise and the solution was stirred for 6 h. The RUB‐15@PZS precipitates were then collected and washed with methanol in triplicate and dried in vacuum at room temperature for 12 h. In the last step, the RUB‐15@PZS was calcined at 700 °C for 4 h in a tubular furnace under an Ar atmosphere, thereby generating the NPS‐GLCs following the removal of RUB‐15 by corrosion with use of NaOH solution.

*Characterization*: Scanning electron microscopy (JEOL JSM‐7800F), TEM, and STEM (Cs‐corrected JEM‐ARM200F microscope 200 kV) were used to examine the morphology and element distribution of the NPS‐GLCs. Tapping mode AFM measurements were carried out on a Bruker Multimode AFM at ambient conditions. The surface area of the NPS‐GLCs was measured on an Autosorb‐1 analyzer at 77 K by the Brunauer–Emmett–Teller (BET) method with use of N_2_ adsorption–desorption isotherms. The U(VI) concentration was measured by the Arsenazo (III) spectrophotometric method by UV–vis spectra photometer (Thermal Scientific Evolution 200) at 650 nm. The competitive adsorption on UO_2_^2+^ + Cs^+^ (or Sr^2+^, Co^2+^, Ni^2+^, and Eu^3+^ ions) binary‐metal systems were measured on the inductively coupled plasma optical emission spectrometry (ICP‐OES) (Thermos iCAP 6300). The XPS spectra of the NPS‐GLCs and UO_2_^2+^‐adsorbed NPS‐GLCs were performed on a Thermo VG RSCAKAB 250X.

*Adsorption Experiments*: The batch adsorption experiments of U(VI) on the NPS‐GLCs were implemented as follows. The suspensions of 10 mg NPS‐GLCs, the U(VI) stock solution, and the background electrolyte solution (10 mmol L^−1^ NaNO_3_) were added together to achieve the desired concentrations. The mixture was kept under magnetic stirring at room temperature for 24 h. The pH in each vial was adjusted by adding certain volumes of 0.001–0.1 mol L^−1^ HCl or NaOH (ionic strength influence was negligible). The above suspensions were stirred for 24 h and withdrawn by filtration (through filter paper, Whatman no. 1), and the concentration of the residual U(VI) in the supernatant was determined. To examine the pH dependence of the adsorption, the UO_2_^2+^ solutions at different pH levels ranging from 2 to 10 were prepared. Uranium had an initial concentration of 200 mg L^−1^, and the *V*/*m* ratio of the batch experiment was 5000 mL g^−1^ (*V* = 6 mL, *m* = 1.2 mg of NPS‐GLCs) at room temperature. For the kinetic sorption studies, the suspensions of 10 mg NPS‐GLCs, 20 mg L^−1^ U(VI) stock solution, and 10 mmol L^−1^ NaNO_3_ were first mixed. The suspensions were then filtered and the filtrates were detected at various reaction times.

The competitive sorption isotherms were investigated at pH of 5.0 ± 0.1 for U(VI) and the competing ions. The selective adsorption performance of the NPS‐GLCs was conducted in the binary solute (U + competing ions) systems under the following conditions: *C*_o_ (U)/*C*_0_ (M) = 1.0, pH = 5.0, *m*/*V* = 200 mg L^−1^, 0.01 mol L^−1^ NaNO_3_, and *T* = 298 K.

The Langmuir adsorption (Equation (1)) model was employed to analyze the experimental data

(1) $$Q_{e}=Q_{m}\;\frac{bC_{e}}{1\;+\;bC_{e}}$$

where *C*_e_ denoted the equilibrium concentration of U(VI) (mg L^−1^), *Q*_e_ referred to the amount of U(VI) adsorbed per unit weight of the adsorbent at equilibrium (mg g^−1^), and *Q*_m_ (mg g^−1^) was the maximum adsorption capacity.

The distribution coefficient (*R*_d_),^38^ which was usually applied to understand the adsorption ability of the adsorbent, was defined as Equation (2)

(2) $$K_{d}=\frac{V}{m}\;\frac{\left(C_{0}-C_{e}\right)}{C_{e}}$$

where *m* was the mass of the adsorbent NPS‐GLCs (g), *V* was the volume of the solution (mL), and *C*_0_ and *C*_e_ represented the initial and equilibrium concentrations, respectively. The pseudo‐second‐order kinetic model was defined as Equation (3)

(3) $$\frac{t}{Q_{t}}=\frac{1}{k\cdot Q_{e}^{2}}+\frac{1}{Q_{e}}$$

where *k* was the pseudo‐second‐order rate constant of adsorption (g mg^−1^ min^−1^), *t* was the adsorption time (min), and *Q*_e_ and *Q*_t_ were the amounts of U(VI) adsorbed (mg g^−1^) at equilibrium and at contact time *t*, respectively. The selectivity coefficient *K*_s_(U/M),^39^ which was described in Equation (4), was introduced to compare the selectivity of the NPS‐GLCs to capture U(VI) from the mixed solution of U(VI) and the competitive ions as follows

(4) $$K_{S}\;\left(\frac{U}{M}\right)=\frac{K_{d,U}}{K_{d,M}}$$

where *K*_d,U_ (mL g^−1^) and *K*_d,M_ (mL g^−1^) are the distribution coefficients of U(VI) and the competing metal ion, respectively.

*Computation Details*: All the structures were optimized by use of the PBE1PBE functional of the DFT methods that were implemented in the Gaussion09 software package. The cc‐pVDZ basis set was used for light atoms, such C, H, O, N, P, S, and so on, whereas the Stuttgart–Dresden–Bonn group (SDB) basis set^40^ was employed for the U atoms. The conductor polarizable continuum model (CPCM)^41^ was employed to mimic the solvation effect. The adsorption energy (*E*_ad_) is calculated as follows

(5) $$E_{\mathrm{ad}}=E\left(\mathrm{G–;X}\right)+E\left(\mathrm{uranyl}\right)-E\left(\mathrm{G–X/uranyl}\right)$$

where the *E*(G‐X), *E*(uranyl), and *E*(G‐X/uranyl) corresponded to the total energies of the defective graphene, the uranyl, and their complexes, respectively.

## Conflict of Interest

The authors declare no conflict of interest.

## Supporting information

Supplementary
