# Supplementary material for: N, P, and S Codoped Graphene‐Like Carbon Nanosheets for Ultrafast Uranium (VI) Capture with High Capacity
Source: Adv Sci (Weinh). 2018 Aug 27;5(10):1800235. doi: 10.1002/advs.201800235 (PMC6193150; doi:10.1002/advs.201800235)
Supplement: Supplementary file 1 — Supplementary [file ADVS-5-1800235-s001.pdf]

Copyright WILEY-VCH Verlag GmbH & Co. KGaA, 69469 Weinheim, Germany, 2018.

ADVANCED SCIENCE
Open Access

Supporting Information

for *Adv. Sci.,* DOI: 10.1002/advs.201800235

N, P, and S Codoped Graphene-Like Carbon Nanosheets for Ultrafast Uranium (VI) Capture with High Capacity

Zhe Chen, Wanying Chen, Dashuang Jia, Yang Liu, Anrui Zhang, Tao Wen, Jian Liu, Yuejie Ai, Weiguo Song,* and Xiangke Wang**

Copyright WILEY-VCH Verlag GmbH & Co. KGaA, 69469 Weinheim, Germany, 2016.

Supporting Information

N, P, and S co-doped graphene-like carbon nanosheets for ultrafast uranium (VI) capture with high capacity

Zhe Chen, Wanying Chen, Dashuang Jia, Yang Liu, Anrui Zhang, Tao Wen, Jian Liu, Yuejie Ai, Weiguo Song* and Xiangke Wang**

Table of Contents

Figure S1. The illustration of plane view and side view of RUB-15.

Figure S2. a) SEM and b) TEM images of RUB-15

Figure S3. XRD patterns of RUB-15.

Figure S4. AFM images of NPS-GLCs..

Figure S5. N_2 adsorption–desorption isotherms of NPS-GLCs. The inset is size distribution curve by DFT method of NPS-GLCs.

Figure S6. The adsorption isotherm for UO_2^{2+} on N, P, and S co-doped carbon bulk materials.

Figure S7. Effect of solid content of NPS-GLCs to the removal percentage of U.

Figure S8 The FTIR spectra of NPS-GLCs and UO_2^{2+} adsorbed NPS-GLCs.

Table S1 Parameters for Langmuir and Freundlich model fits at 298 K of the UO_2^{2+} adsorption on NPS-GLCs.

Table S2 The removal percentages of UO_2^{2+} and the metal ions in the competing adsorption

Figure S9 The structure of the G-X (or G-X') complexes.

Figure S10 The optimized structures of the G-X'/ Uranyl (U) complexes. X' were the pyridinic N and S existed at the edge of the hole on graphene.

Table S3 The calculated total electronic energies (in hartree) and adsorption energies (kcal/mol) by DFT method.

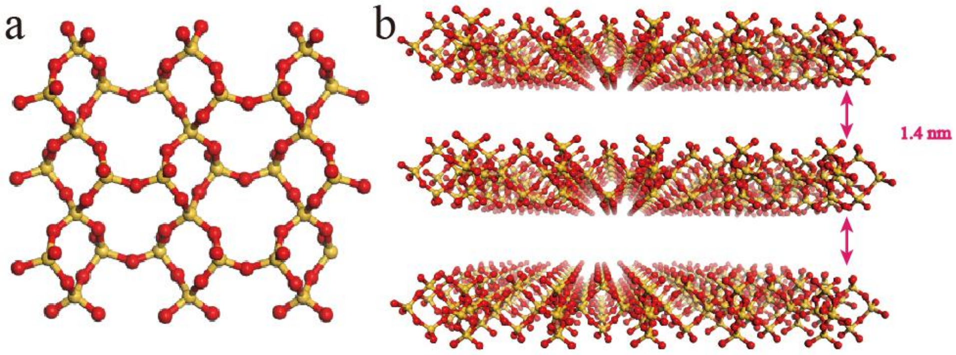

Figure S1. The illustration of plane view and side view of RUB-15.

Figure S1 showed the planar and side view of the aluminium-free layered silicate RUB-15, which had zeolitic framework compose of a halved sodalite cage. The layer spacing is 1.4 nm.

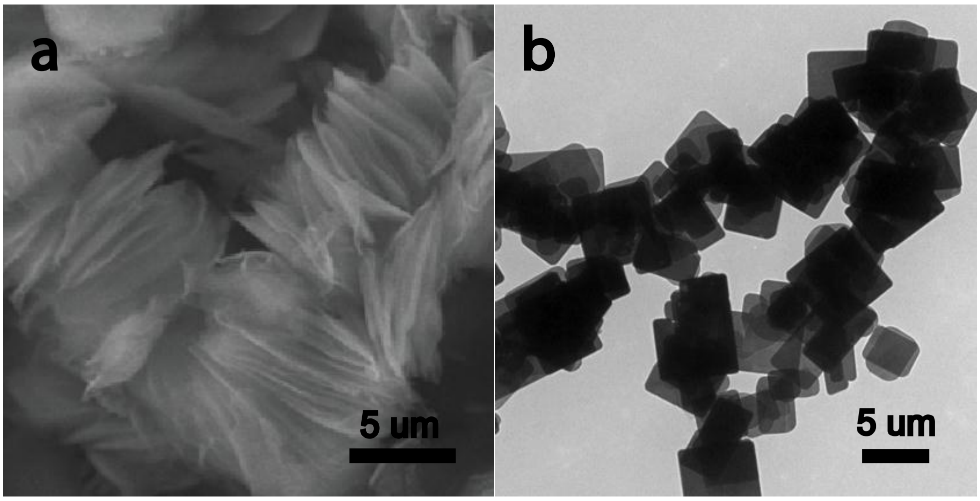

Figure S2. a) SEM and b) TEM images of RUB-15

Figure S2 were the SEM and TEM images of RUB-15. RUB-15 were thin and flexible nanosheets, with the length of 5-10 μm and thickness of less than 100 nm.

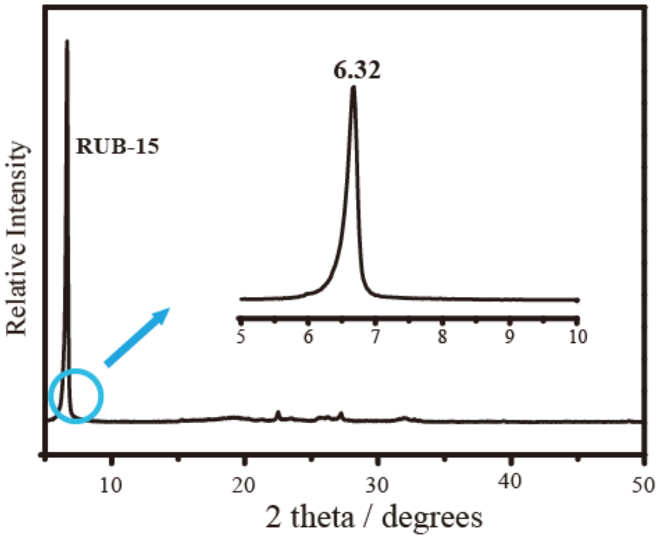

Figure S3. XRD patterns of RUB-15.

The XRD pattern of RUB-15 showed a strong signal at 2θ = 6.32°, suggesting the basal spacing of 1.40 nm. The sharpness of the reflections con-firmed the purity of RUB-15.

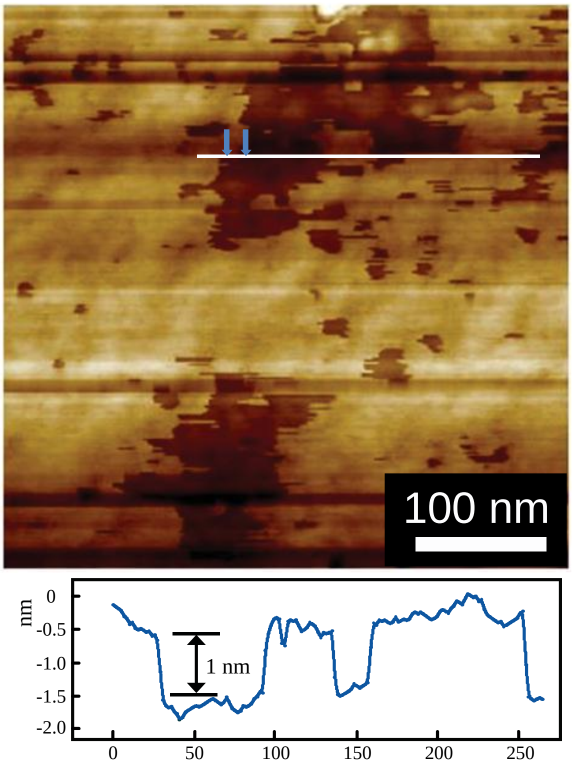

Figure S4. The AFM image of NPS-GLCs and the cross-section height profile.

Figure S4 displayed the atomic force microscopy (AFM) images of NPS-GLCs, proving the morphology of few layers of ultrathin graphene-like membranes. The cross-section height profile revealed a small thickness of 1.0 nm, that was, two or three graphitic layers, which agreed well with the TEM images.

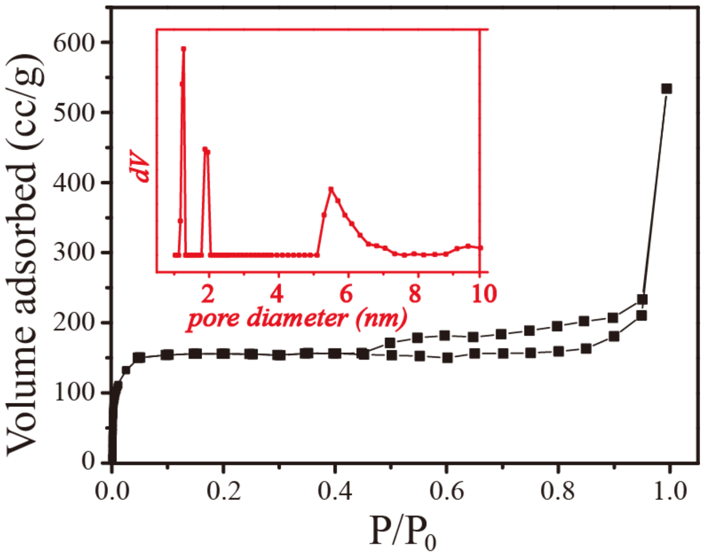

Figure S5. N2 adsorption–desorption isotherms of NPS-GLCs. The inset is size distribution curve by DFT method of NPS-GLCs.

The porous distribution of the NPS-GLCs was determined by the N_2 adsorption-desorption isotherms, and displayed in Figure. S5. The sharp increase of the adsorbed volume in the low-pressure region and the hysteresis loop in the medium pressure region were assigned to the typical type-I and type-IV isotherms, thereby proving the existence of micropores and mesopores in the NPS-GLCs. The Brunauer–Emmett–Teller (BET) specific surface area was calculated as 460.3 m^2/g. The pore size distribution simulated by the density functional theory (DFT) showed three peaks at 1.2 nm, 2.0 nm, and 5.6 nm (Figure. S5 inset). The sharp peak around 1.2 nm was consistent with the 1.0 nm layer spacing observed on the TEM image. The pore distribution at 2.0 nm and 5.6 nm might come from the accumulation and stacking of the NPS-GLCs nanosheets. The relatively high surface area and porous structure was very beneficial for adsorption as it supplied abundant active sites to combine with adsorbates.

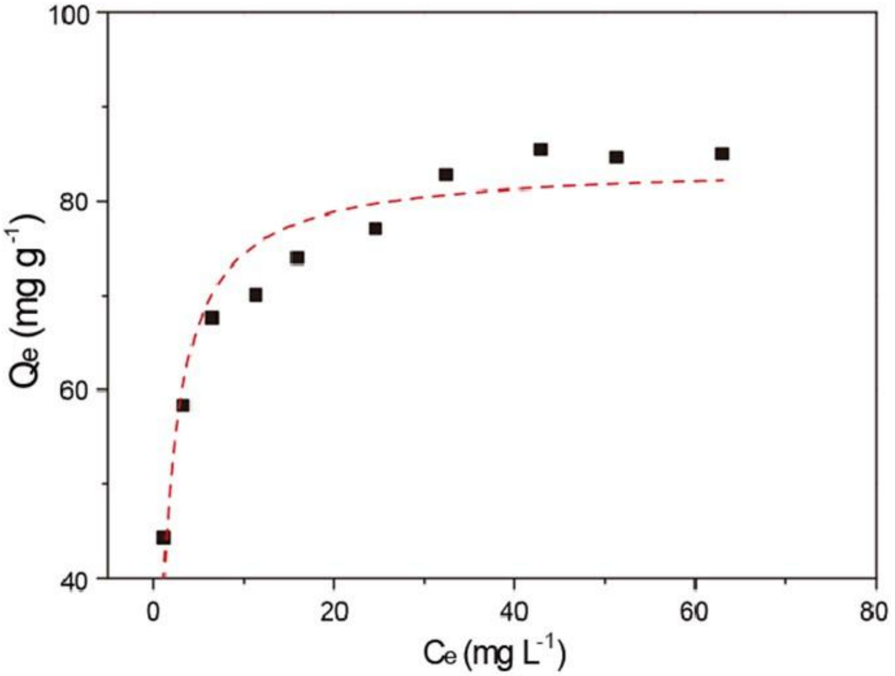

Figure S6 The adsorption isotherm for UO_2^{2+} on N, P, and S co-doped carbon bulk materials.

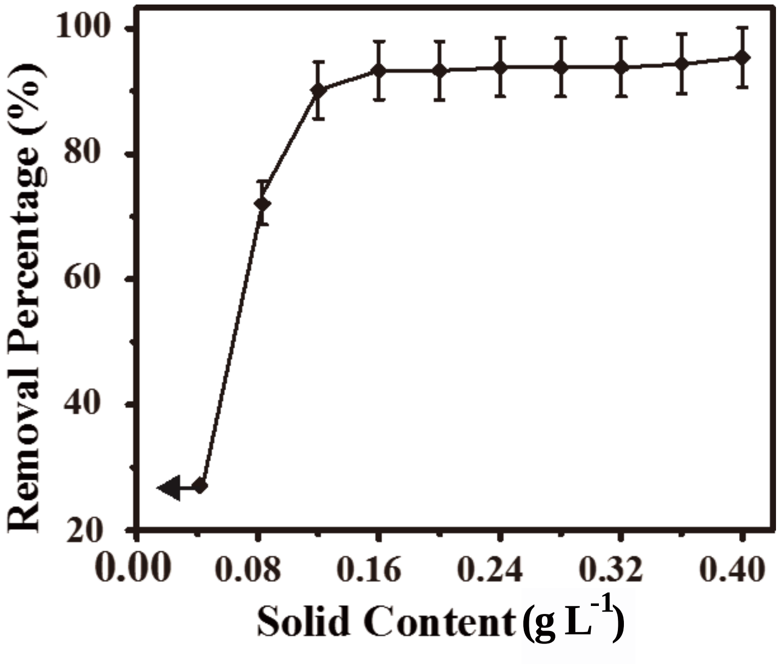

Figure S7 Effect of solid content of NPS-GLCs to the removal percentage of U.

The effect of the ratio of solution volume (V) to adsorbent mass (m) on the removal efficiency of uranium was tested. As shown in Figure S7, the dependence of U(VI) sorption on NPS-GLCs at different solid contents were investigated at T = 298 K and

pH = 5.0. With the increase of NPS-GLCs content from 40 mg L^{-1} to 400 mg L^{-1}, the sorption percentages increased obviously from 27.2 % to 96.0 % for U(VI). Inspired by it, the solid content of NPS-GLCs was selected as 200 mg L^{-1} in the batch experiments.

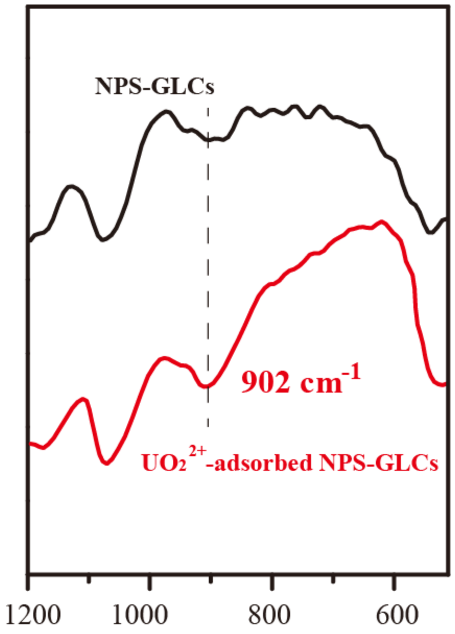

Figure S8. The FTIR spectra of NPS-GLC and UO_2^{2+} adsorbed NPS-GLC.

Table S1 Parameters for Langmuir and Freundlich model fits at 298 K of the U(VI) adsorption on NPS-GLCs

Langmuir model	Q_{max}	K_L	R^2
	294.16	0.332	0.993
Freundlich model	K_F	n	R^2
	105.64	3.66	0.765

The Langmuir model (equation 1):

$$Q_e = Q_m \frac{bC_e}{1 + bC_e} \qquad (1)$$

The Freundlich model (equation 2):

$$q_e = K_F c^{1/n} \qquad (2)$$

Table S2 The removal percentages of UO_2^{2+} and the metal ions in the competing adsorption

	Cs^+	Sr^{2+}	Co^{2+}	Ni^{2+}	Eu^{3+}
UO_2^{2+} Removal percentage	84.7	83.9	81.5	82.6	84.2
Removal percentage of Metal ions	3.4	4.8	11.9	10.0	19.8
$\mathbf{K_S}\,\frac{\mathbf{U}}{\mathbf{M}}$	157.3	103.4	32.6	42.7	21.6

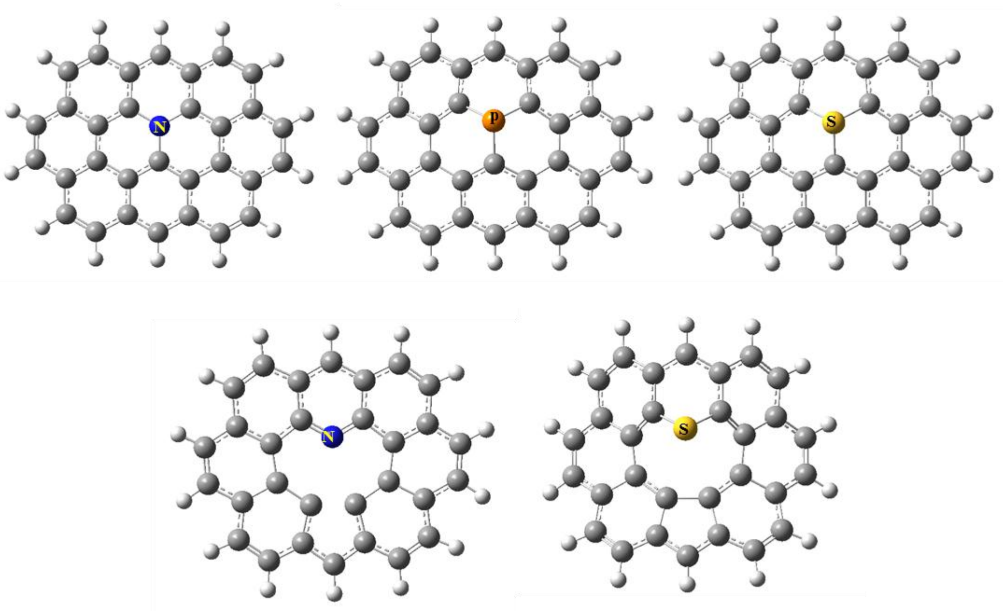

Figure S9. The structure of the G-X (or G-X') complexes.

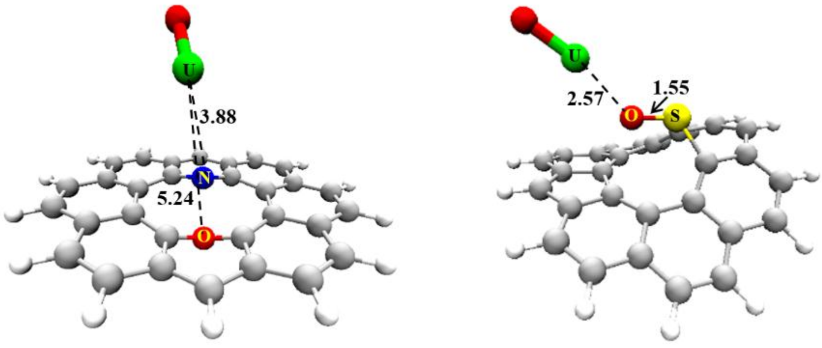

Figure S10. The optimized structures of the G-X'/ Uranyl (U) complexes. X' were the pyridinic N and S existed at the edge of the hole on graphene.

The effect of the holes and defects generated along with the graphitization of PZS was also considered. Figure S10 presented the UO_2^{2+} interaction with the pyridinic N and S existed at the edge of the hole. In both situations, the underlying substrate grabbed the oxygen atom of uranyl, which indicated that the high surface energy sites and reducibility derived from the defects carbon atoms on the porous graphene also benefitted the UO_2^{2+} interaction on the NPS-GLCs.

Table S3 The calculated total electronic energies (in hartree) and adsorption energies (kcal/mol) by DFT method.

Reactant	hartree	Reactant	hartree	Product	hartree	E_{ad}(kcal/mol)
G-N	-1243.2801	$UO_2(H_2O)_5$	-1008.967145	G-N/ Uranyl(U)	-2252.28256376	22.16
G-N	-1243.2801	$UO_2(H_2O)_5$	-1008.967145	G-N/Uranyl(O)	-2252.28971996	26.65
G-P	-1529.755266	$UO_2(H_2O)_5$	-1008.967145	G-P/ Uranyl(U)	-2538.73799664	9.78
G-P	-1529.755266	$UO_2(H_2O)_5$	-1008.967145	G-P/Uranyl(O)	-2538.7532111	19.33
G-S	-1586.537029	$UO_2(H_2O)_5$	-1008.967145	G-S/ Uranyl(U)	-2595.51575852	7.27

The elution experiments

After the adsorption of U(VI), three method had been used to regenerate the NPS-GLCs by applying excess KCl solution (0.2 mol/L),HCl solution (0.2 mol/L) or pure ethanol. The results showed that NPS-GLCs could not be regenerated completely by the above-mentioned three eluents, as the removal percentage of U(VI) at the 2nd run decreased to 4.0%, 19.1%, and 4.5% , respectively, comparing with the first run. The regeneration capacity of NPS-GLCs was not excellent but was reasonable here. As described in the manuscript, the strong covalent bonds between P-O-U and S-O-U as well as the reducibility from the defects and holes on the NPS-GLCs were the key active sites for UO_2^{2+} fixation, proved by the XPS analysis and the DFT calculations. Different from usual adsorption mechanism such as ion-exchange, surface complex and van der Weals forces, the covalent bonds between P-O-U and S-O-U were hard to break by K^+ ions or oganic solvent. The elution of H^+ showed better regeneration efficiency (19.1% capacity was regenerated), which might be derived from their higher binding ability to form H-O-U and then the UO_2^{2+}separated from NPS-GLCs. As a result, the effective eluent for NPC-GLCs were still under searching.
